# A tool for exploring space-time patterns : an animation user research

**DOI:** 10.1186/1476-072X-5-35

**Published:** 2006-08-29

**Authors:** Patrick J Ogao

**Affiliations:** 1 Faculty of Computing & Information Technology, Makerere University, P.O. Box 7062, Kampala, Uganda

## Abstract

**Background:**

Ever since Dr. John Snow (1813–1854) used a case map to identify water well as the source of a cholera outbreak in London in the 1800s, the use of spatio-temporal maps have become vital tools in a wide range of disease mapping and control initiatives. The increasing use of spatio-temporal maps in these life-threatening sectors warrants that they are accurate, and easy to interpret to enable prompt decision making by health experts. Similar spatio-temporal maps are observed in urban growth and census mapping – all critical aspects a of a country's socio-economic development. In this paper, a user test research was carried out to determine the effectiveness of spatio-temporal maps (animation) in exploring geospatial structures encompassing disease, urban and census mapping.

**Results:**

Three types of animation were used, namely; passive, interactive and inference-based animation, with the key differences between them being on the level of interactivity and complementary domain knowledge that each offers to the user. Passive animation maintains the view only status. The user has no control over its contents and dynamic variables. Interactive animation provides users with the basic media player controls, navigation and orientation tools. Inference-based animation incorporates these interactive capabilities together with a complementary automated intelligent view that alerts users to interesting patterns, trends or anomalies that may be inherent in the data sets. The test focussed on the role of animation passive and interactive capabilities in exploring space-time patterns by engaging test-subjects in thinking aloud evaluation protocol. The test subjects were selected from a geoinformatics (map reading, interpretation and analysis abilities) background. Every test-subject used each of the three types of animation and their performances for each session assessed.

The results show that interactivity in animation is a preferred exploratory tool in identifying, interpreting and providing explanations about observed geospatial phenomena. Also, exploring geospatial data structures using animation is best achieved using provocative interactive tools such as was seen with the inference-based animation. The visual methods employed using the three types of animation are all related and together these patterns confirm the exploratory cognitive structure and processes for visualization tools.

**Conclusion:**

The generic types of animation as defined in this paper play a crucial role in facilitating the visualization of geospatial data. These animations can be created and their contents defined based on the user's presentational and exploratory needs. For highly explorative tasks, maintaining a link between the data sets and the animation is crucial to enabling a rich and effective knowledge discovery environment.

## Background

Computer animations have apparently been a subject of great interest among the computer graphics and mainstream media enthusiasts. Lately, it has also featured prominently in health and geoinformatics [1-3]. However, the necessary theory and functionality to apply it in exploratory environments in these animations has been lacking or not yet fully developed. The techniques at our disposal in animation were still in their infancy in terms of the types of functionality use and adaptability to geospatial data sets. Initially most of the development in animation functionality came directly from the media, especially from video and film technology as can be seen in the earlier passive animation, in which viewers had to be content with the pre-assembled key-frame animation no matter how intriguing or irrelevant the contents were.

In view of these trends, researchers have focused on providing tools that will enhance investigation amongst geo-scientists in geospatial data exploration through emphasis on the purposeful exploration and search for patterns among a given data set [4,5].

A key issue with all these developments is that visualization should not just be limited to enabling the process of seeing patterns and relationships in geospatial data, but rather to envisage manipulating geo-structures to search, filter, control level of detail, reorganize, change, and derive new useful information. The context of using visualization tools should extend to encompassing individual private exploration as well as collaborative exploration in distributed environments.

## Results

For every evaluation, there is the need to ensure that the results attained are reliable and valid [6]. The users' cognitive structures and processes also go a long way to verifying the exploratory design model [7].

**Figure 1:**
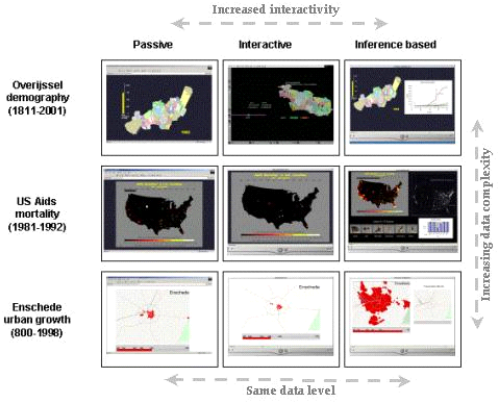
**Case Studies**. The case studies data sets and the type of animation produced for the evaluation test. (Overijssel demography and Enschede urban growth data used with permission from ITC, Division of cartography and visualization, Enschede, The Netherlands. US Aids mortality data used with permission from   the Center for International Earth Science Information Network (CIESIN), US National Institutes of Health's National Cancer Institute, and the National Library of Medicine. 1995. AIDS Data Animation Project. Palisades, NY: CIESIN, Columbia University.

Three case studies that involve data sets with varying levels of complexities were selected (Figure 4). Overijssel demographic changes between the years 1811 – 2001, US Aids Mortality for the period 1981 – 1992, and the growth of Enschede town between the years 800 to 1998 (Figure 1). Two main groups of test subjects emerge out of this. Group A are those who use each type of animation with the same data sets. A total of 22 test subjects made up this group. Group B test subjects are those who use each type of the animation to explore different data sets. A total of 13 test subjects made up this group.

Tests undertaken by Group A test subjects reveal the need for tools for the user to interact with the animation during display. Results also show that out of the 35 test subjects of the two main evaluation groups (Groups A & B), a total of 30 expressed the need to interact with the animated map. Comparisons made in Group A, between the interactive and inference-based animation highlighted details of the interactivity desired to improve the interface. A finer temporal interval or ability to choose the temporal resolution between the animation frames, frame rate control, basic media play, stop controls, data access capabilities, orientation and navigation capabilities were among the necessary and commonly listed tools.

Of the 35 test subjects (Groups A & B) using the inference-based animation, 22 used the complementary inference view to enhance their understanding of the case study. Test subjects were quick to pursue the hints provided by these views. The views seem to direct test subjects into an active exploratory status compared to when they use the other types of animation. 30 test subjects from both Groups A and B used or showed the need for the generic operators of: identify, locate, compare or associate.

**Figure 2:**
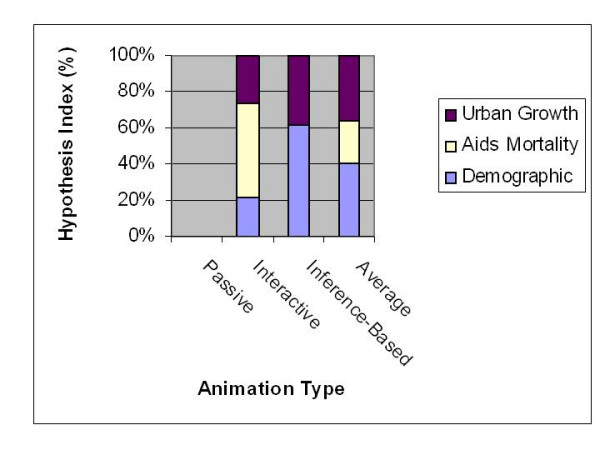
**Contribution of each type of animation to hypothesis formulation (Group A and B)**. A hypothesis index is the ratio of the number of hypothesis formulated to the number of test subjects involved In the test.

Group A test subjects formulated a total of 9 hypotheses (Figure 2). There were 3 hypotheses using interactive, and 6 for inference-based animation). These results also tell something about the data sets used. They confirm the varying level of complexity amongst the three case studies. No hypothesis counts were recorded for test subjects using passive animation. Group B test subjects formulated a total of 12 hypotheses as shown in Figure 2.

**Figure 3:**
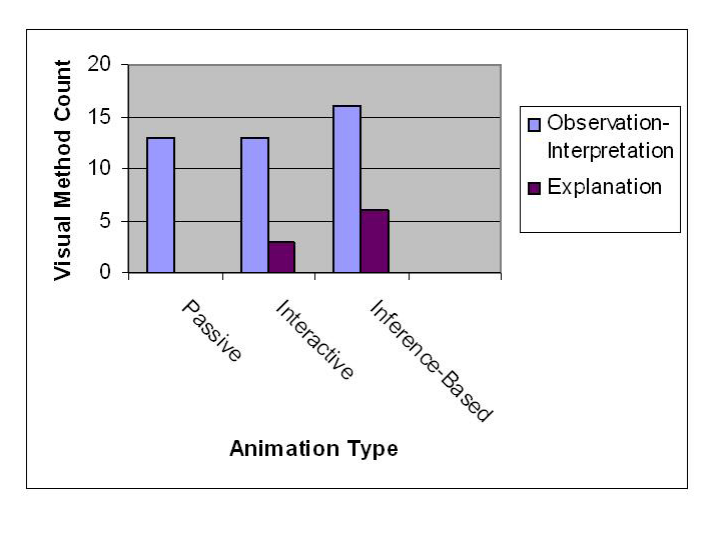
**Comparing counts between stages in the visual method**. The figure shows a graph comparing the counts obtained from a combined count of observations and interpretations to that of explanations. These are plotted for the three types of animation used. Combining the observation and interpretation counts was necessitated because of the difficulties encountered in making distinctions between the two stages.

The visual methods stages that were considered were: observation, interpretation and explanation. This in essence translates into the "seeing that – reasoning why" phases in visualization [8]. Observation as used in the evaluation only gives the visual description of the representation in the specific animation. Visual description is characterized here by the test subject's use of the sensory input that translates into a description that utilizes perceptual schemata. Observations thus would focus on the typical graphic marks that are represented (Figure 3).

Finally in explanation and which we also term the hypothesis formulation phase, test subjects are able to hypothesize when they encounter meaningful patterns exhibited in the animation. This they do by utilizing their knowledge about the domain or case study involved and hypothesizing about the cause of the patterns observed.

## Discussion

Animation parameters that include the frame rate speed, frame sequence, and viewing perspective affect users differently. Results showed that users want to be in control of the dynamic display. They want to play, stop and pause the animation at their own pace and rate. Inability to interact results in frustration as was evident in the tests. Test subjects were prompted (or desired) to vary the animation speed depending on where their attention was focused. Using the Overijssel animation for example, test subjects found the frame rate appropriate when they had their attention focused on the 3-D municipality boundary-population map. The moment that they expressed interest in knowing when (time) observed changes occurred they found the frame rate too fast. This pinpoints the need to optimize on animation production.

Employing interactivity in animation is also a convenient and intuitive way that visualization operators (identify, locate, compare & associate) can be implemented in the animation environment. Observing users move the mouse over features and click the mouse expecting to get some feedback on what they are seeing reinforces the need for tools to enable visualization operations. Related to this is the expression of the need to access further data. Users want to confirm their initial thoughts and explanations with the observed patterns and trends. To do this they expressed the need for tools to give them access to the original or related data sets. Tools here should facilitate selection of a specified geographic layer and attribute tables.

The explanation that can be attributed to the equal rating in the formulated hypothesis between the passive and interactive animation points to their possible equal performance in exploration stages specifically when the data levels in each of the case studies is maintained at the same level. Whereas these restrictions were solely to avoid data related bias, we are of the opinion that interactivity does more than just control say the media control tools (as was the case in these tests). Their exploratory scope is unlimited when used for accessing the data behind the display.

The results also showed that, test subjects using the inference-based animation attained a higher hypotheses yield. In general, the inference-based animation environment facilitated the formulation of a higher number of hypotheses in both groups. The hints or information that these views provided seem to automatically challenge users to want to explore them further. It is vital to mention here that test subjects were under no obligation to pursue these hints. They could on their own initiate questions worth pursuing. Similarly these additional views did not have any extra information compared with what was provided in passive and interactive animation. They basically provided another perspective of the main view, only in this case they narrowly focused on the patterns that may be of interest to the users.

The distinct uses that each of the three types of animation are subjected to are crucial in exploration. This highlights the importance of each type of animation in the exploration process. Test subjects in Group B who had to confront new study cases in each evaluation session went through an initial time period of no interactivity (either through intentions in passive animation or actions as with interactive animation). This was followed by a time period when they interactively engaged the animation (as in interactive animation). Therefore in inference-based animation, test subjects went through all the elements or stages of passive and interactive animation. Thus the inference-based animation inherits the interface and use characteristics that are incorporated within both the passive and interactive animation.

There is a correlation between the way the three types of animation are used and the visual methods that users employ. Passivity is a trait that characterizes the early stages of visual exploration. The use of passive animation seems to be able to contribute to the visual method of observation and partly to interpretation. These visual methods are evoked and used iteratively during the entire exploratory period. Evoking interactivity as when using interactive and inference-based animation links the already observed and/or identified features in the observation and interpretation stages to the explanation stage where hypotheses are formulated.

A similar pattern occurs when the underlying cognitive structures and processes of a user are traced. The initial observation stages as explained earlier on highlighted the many observations that users could make. The test subject's initial pursuits or expectations were weighed against the final outcome of the interpretations they made of the case study (data sets explored). The hypothesis formulated was characterized by the novelty of the observed patterns. By this we mean; the majority of hypotheses were centered on describing and hence explaining the unique patterns that were inherent in the data. The majority of hypothesis were not centered around describing the normal patterns, but only those that seemed abnormal. The point is that, the visual method of observation and interpretation seem to dominate the stages before any unique or spatial structure with appropriate pre-inventive properties is discovered. Once that is attained, the explanations seem to follow.

## Conclusion

The fundamental observation that guided this work is that the environment in which animation are used at present does not support the exploratory pursuits that geoscientist undertake. These environments lack or have no functionalities to enhance the exploration process. Finding out their true exploratory performance is inhibited by uses that intuitively favour embedding graphical displays with interactive and dynamic tools as a qualifying trait for exploration. This seem to crop up from the continual improvement and proliferation of graphics hardware for workstations and personal computers, where performance is characterized by fast, high quality graphics displays coupled with highly expressive interactive input devices to achieve real-time visualization. But most importantly, no research had been undertaken to define a nature or set of properties that would qualify animation environments as being self sufficient for undertaking exploratory tasks. We thought that defining these properties would not only help in designing animation functionalities that steer exploration, but also would help to evaluate the resulting end-products. Exploratory products in this sense, are then not just abstract knowledge pop-ups, but rather are result of the purposeful and skilful use facilitated by geoscientists using visual functionalities to construct new knowledge. This knowledge has a valid construct in that, the results obtained can be generally perceived as unique, sensible and practical even by others not involved with the tasks.

In this regard, we are confident that this paper is a first step towards formalizing the design and uses of temporal animation. At a glance, what one sees emerging is a cross-disciplinary approach that transcends the disciplines in cartography, cognitive science, human computer interaction (HCI), computer animation, artificial intelligence and geoscience application domains. This may seem out of place in research that is undertaken within mainstream realms of cartography. However, what it pinpoints is that dealing with such a subject as animation calls for cross-disciplinary collaborative research initiatives where work and results can be shared and yet still maintain the prevailing disciplinary focus.

Certain issues emerged while pursuing this study. First, the paper did not account for certain aspects of the dynamic variables, which we think, may contribute significantly to exploratory tasks [9-12]. Dynamic variables may influence one's ability to detect geospatial dynamics. As yet, the outcome from research dealing with dynamic variables is still very scanty. An improvement to the optimized animation as used in this paper could account for their effects on the test subject's performance.

Second, our use of re-expression to provide an alternative view of the transformed data did not specifically distinguish between the possible re-expressions that can be attained. Though we used reordering in the inference-based animation, we think it will be appropriate to specify and investigate the contribution of the many ways that re-expressions can be effected. Part of our limitation was due to the focus on temporal aspects of animation. But the value of these methods could well be realized even with the non-temporal and successive build-up animation.

Third, temporal animation are highly suitable for implementation in a temporal GIS (TGIS) environment. To date, the development and implementation of geographical visualization (GVis) and TGIS has yet to be realized. Thus for animation and the subsequent functionalities as defined in this paper, we anticipate initial implementation at a prototype level. The implementation should enable users to visualize the data and focus on what is relevant, thereby transcending the presentational realms of visualization. Technical considerations on the implementation should ensure that the animation is not tightly coupled to other tasks in the system, since this may slowdown the system's performance. Implementing exploratory temporal animation is synonymous with current work that seeks to integrate GVis, GIS and knowledge discovery in databases (KDD) into comprehensive systems that have interactive visual displays, temporal geospatial operations and data mining capabilities [13].

Fourth, imbuing animation with domain intelligence is an area of study that has great significance for geo-applications dealing with disaster prevention, early warnings and emergency fields. These studies require that the phenomena and information is dynamically visualized in real time to enable a timely response by management teams. It requires dynamic visualization systems that not only utilize historical data sets, but also extrapolate data to forecast a future visual scenario of the phenomena. These intelligent traits could be utilized in research dealing in intelligent autonomous agents. In particular, we single out methods that provide a finite and succinct way of representing uncertainty in the features world and the numerous possible decision alternatives. This makes it possible to imbue features with knowledge, monitor the status and contents of the world in which they and track any possible changes that may occur.

Lastly, the use of animation should of course be within the confines of acceptable interactivity levels, graphic mix and dynamics, since the rich and rapid graphic sequences of graphics can overwhelm the eye-brain system. Future research studies should look at the influences of varying ranges of graphics and interactivity levels on the user's performance. These aspects if not controlled may have adverse cognitive influences thereby affecting learning, decision making and problem solving.

## Methods

Thinking-aloud protocol and retrospective testing techniques using test data sets taken from the realms of demography, epidemiology and urban studies were used. Thinking-aloud protocol is a method for gathering information about actual use of a system [6]. Test the subjects are observed as they interact with the system. By verbalizing their thoughts, feelings, and opinions while interacting with the system, the method is capable of capturing a wide range of cognitive activities.

Since the test sessions using the thinking-aloud method was recorded on videotape, more information could be gathered by reviewing the tapes together with the test subjects. Test-subjects were asked to expound on specific questions regarding their behaviour during the exercise.

**Figure 4:**
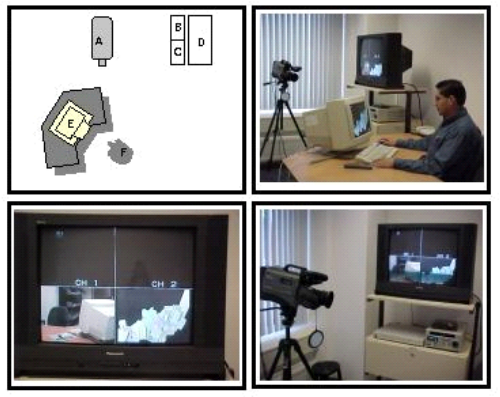
**Set up of the usability laboratory**. Top picture shows the plan and pictorial view of the laboratory equipment A – video camera, B – digital quad unit, C – VCR, D – TV monitor, E – the Computer and 17' monitor, and F – the test subject. Bottom left is the TV monitor with a 4-part split view. Bottom right shows the video camera, the digital quad unit right and the VCR.

A laboratory room was dedicated for the evaluation exercise. The need was to have an isolated, quiet and yet spacious room to take all the needed equipment. A plan and the corresponding pictures of its constituents are shown in Figure 4. The digital quad unit simultaneously takes in signals from the video camera, wireless microphone attached to the test subject, and the computer. The signals are temporally synchronized and appear in a 4-part split view in the TV monitor. The TV image consists of a perspective view of the test subject. With this one sees the facial expressions and a considerable amount of the body movements. Also on view are all the activities (mouse pointer, application scenes) on the computer monitor. The image as seen on the TV monitor is recorded on to a videotape in the video camera recorder (VCR). Other systems specifications are highlighted in [14].

The geospatial data available to all the animation environments are maintained at the same level of information density and complexity. This means that the symbols were easily distinguishable and any relationships between them could be found. The complexity also varied from simple 2- to 3-dimensional geospatial data sets. Also, since the test was concerned with evaluating the performance of different types of animation despite each type being used by different data sets, the information conveyed in each animation was maintained at the same level. This eliminates the bias related to offering varying levels of data and information details to users. Test subjects have to undertake the same exploratory task within the prescribed time. The animation constants or the dynamic variables are initially set to the same levels. For example, on playback the frame rate, orientation and perspectives of each of the types of animation are the same. Of course depending on the type of animation, users may when performing tasks, change any of these variables as they may deem fit. The structure of patterns exhibited in each case study is unique – e.g. clusterly and overly distributed, random and centric (showing patterns increasing outwardly from a central location. They help avoid bias due to obstruction, confined attention or concentration resulting from a restricted area of activity.

35 test subjects participated in the evaluation exercise. They were all unpaid postgraduate students volunteers with 60% in geoinformatics domains. 20% were from urban planning and the rest from geology, population studies, forestry, computer science and natural resource management. 80% of test subjects were in the age group 25–30 years, with the rest being at age group, 31–40 years. A third of the participants were female. Being postgraduate students, they have spent an average of 5 years in professional work in their relevant geo-disciplines.

The ability to read and interpret geospatial representation was a necessary requirement for the participants, since emphasis was at the animation interface and functionality level. All the test subjects indicated that they were familiar with animation, though none of them had used them practically in their professional work. The test subjects are therefore regarded as being homogeneous based on their backgrounds (geo-expertise), present career interests or study status, their expressed interest in the use of maps and visual products in general.

The test procedure was to prepare and enable the test subject to undertake a free and unconstrained visual exploration in any of the case studies discussed earlier. This was done in three stages: a trial session, the actual test that utilized the thinking-aloud protocol and lastly a retrospective test.

This is an introductory test that lets the test subject get accustomed to the test environment. The subjects have a few of their professional details taken. This includes their professional designations, years of work experience, experience with maps and animation among other details in a typical informal chart. Next, they are exposed to the laboratory room and equipment. They are introduced to an animation previously compiled using some test data (not related to the data used in the main test). They get the opportunity to play with the animation controls of the QuickTime player, and the Cortona 3.1 VRML client.

The practice session is crucial since it prepares and places the test subjects on a level starting position as regards familiarization with animation and its associated tools. They also get sufficient time to get acquainted with the test environment.

Using the mentioned test animation, subjects are lead through a trial session. They are trained to think aloud along a line of thoughts that help bring out the subject's initial observations, interpretations and animation functionality.

Each test subject was expected to formulate the best claim or conclusion about the phenomenon depicted by the animation. This was done by locating indicators consisting of such words as, therefore, thus, so, as a result, consequently and my conclusions are that. Based on the realized conclusion, it was then more sensible to analyze the arguments. This involved looking for premises that directly support the conclusion. The premises consisted of, supporting arguments, evidence, assumptions, authority and explanations. They could be traced from use of such words as, since, for, supposing that, given that, because and assuming that. In essence what the foregoing translates to is the tracing of the three inferences of deduction, induction and abduction, from the test subjects accounts during the thinking aloud protocol.

On the visual method adopted during the exploration task, observation entail extracting test subjects visual descriptors (involving a conceptual representation of the display), interpretation (identifying meaningful patterns, trend or anomalies), and explanation (hypothesizing the causes of the phenomenon in the interpretation stage).
